# Supplementary material for: DNA Damage Repair Classifier Defines Distinct Groups in Hepatocellular Carcinoma
Source: Cancers (Basel). 2022 Sep 1;14(17):4282. doi: 10.3390/cancers14174282 (PMC9454479; doi:10.3390/cancers14174282)
Supplement: Supplementary file 1 [file cancers-14-04282-s001.zip › cancers-1878543-supplementary.pdf]

Supplemental Table S1. DNA repair genes by pathway

Pathway	Error Propensity	Genes
Mismatch Repair	Low	EXO1, MLH1, MLH3, MSH2, MSH3, MSH6, PMS2
Nucleotide Excision Repair	Low	ERCC1, ERCC2, ERCC3, ERCC4, ERCC5, ERCC6, LIG1, LIG3, RAD23B, XPA, XPC, XRCC1
Trans-Lesion Synthesis	Low	MAD2L2, POLH, POLI, POLK, RAD18, REV1, UBE2A, UBE2B, USP7
Fanconi Anemia	Low	BTBD12, C17orf70, C1orf86, FANCA, FANCB, FANCC, FANCD2, FANCE, FANCG, FANCI, FANCL, FANCM, MTMR15, PALB2, UBE2T, USP1
Base Excision Repair	Low	APEX1, FEN1, NEIL1, NEIL2, NEIL3, OGG1, PCNA, PNKP
Nucleotide metabolism	Low	RNASEH1, RNASEH2B, RRM1, RRM2, RRM2B, TK1
Template Switch	Low	SHPRH, ZRANB3
Poly ADP Ribose Polymerases	Low	C12orf48, PARP1, PARP2, PARP3, PARP4, PARP9
Checkpoint	High	ATM, ATR, CHEK1, CHEK2, CLSPN, HUS1, HUS1B, MDC1, RAD1, RAD9A, RAD9B, RAD17, TIMELESS, TIPIN
DNA replication factors/Cell Cycle	High	AURKA, AURKB, CCNA1, CCNA2, CCNB1, CCND1, CCND2, CCND3, CCNE1, CDC25A, CDC25B, CDC25C, CDC45, CDC6, CDKN1A, CDKN1B, CDKN2A, CDT1, DDB1, DDB2, E2F1, E2F2, E2F3, E2F4, E2F5, E2F6, E2F7, GMNN, MAX, MCM10, MCM7, MYC, PHB, PLK1, POLD3, POLD4, POLE4, PPP1R12A, RB1, RBL1, RBL2, RFC2, RFC3, RFC4, RFC5, RPA1, RPA2, RPA3, WEE1
Homologous Recombination	High	BARD1, BLM, BRCA1, BRCA2, BRIP1, DNA2, FAM175A, FBXO5, MUS81, RAD51, RAD51AP1, RAD51C, RAD51L1, RAD51L3, RAD54B, RAD54L, RAD54L2, RBBP8, RNF138, TOP3A, TOP3B, TOPBP1, UIMC1, XRCC2
Nonhomologous End Joining	High	DCLRE1C, DNTT, LIG4, MRE11A, NBN, NHEJ1, POLB, POLL, POLM, POLQ, RAD50, RIF1, RNF168, RNF169, RNF8, TP53BP1, XRCC4, XRCC5, XRCC6
Mitosis/Spindle Assembly Checkpoint	High	BUB1, BUB1B, BUB3, CDC20, CDH1, ESPL1, MAD1L1, MAD2L1, PTTG1, TRIM69
Repair/Replication DNA Polymerases	High	POLD1, POLE
APOBEC		APOBEC1, APOBEC3A, APOBEC3B, APOBEC3C, APOBEC3D, APOBEC3F, APOBEC3G, APOBEC3H
Cancer Testes Antigens		HORMAD1, MAGEA1, MAGEA4, MAGEA6, MAGEA10

Supplemental Table S2. p53 and HRD-related features in HCC stratified by DNA repair groups.

		1 - Low	2 - High	p-value
**n**		**216**	**158**	
**p53 status**				
	p53 mutant	45 (24.6)	138 (75.4)	<0.001
	WT	171 (89.5)	20 (10.5)	
**TP53 mutation status**				
	TP53 mutation	41 (36.6)	71 (63.4)	<0.001
	WT	175 (66.8)	87 (33.2)	
**HRD status**				
	HRD High	48 (36.1)	85 (63.9)	<0.001
	HRD Low	153 (71.5)	61 (28.5)	
**HBV status**				
	Negative	146 (67.3)	71 (32.7)	<0.001
	Positive	66 (43.7)	85 (56.3)	

Supplemental Table S3. p53 and HRD-related features in HCC stratified by Low-repair subgroups.

		L1	L2	L3	p-value
**n**		**86**	**56**	**74**	
**p53 status**					
	p53 mutant	0 (0.0)	26 (57.8)	19 (42.2)	<0.001
	WT	86 (50.3)	30 (17.5)	55 (32.2)	
**TP53 mutation status**					
	TP53 mut	4 (9.7)	12 (29.3)	25 (61.0)	<0.001
	WT	82 (46.9)	44 (25.1)	49 (28.0)	
**HRD status**					
	HRD High	8 (16.7)	18 (37.5)	22 (45.8)	0.002
	HRD Low	69 (45.1)	35 (22.9)	49 (32.0)	

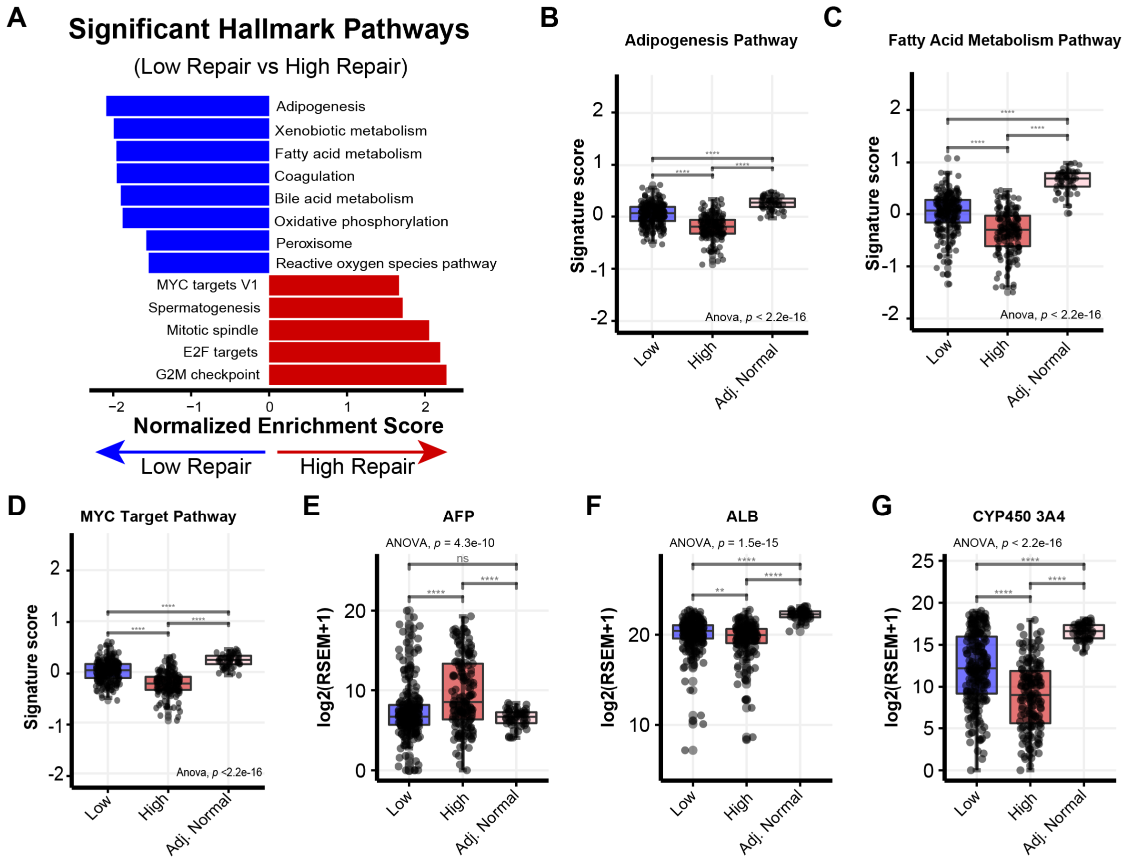

Supplemental Figure S1. Low-repair samples display expression of genes involved in liver function. (A) Gene Set Enrichment Analysis was performed Low-repair (blue) vs High-repair (red) using the Hallmark gene sets. Significant pathways were plotted by normalized enrichment score (NES). One-way ANOVA *p*-value is displayed. (B) Adipogenesis, (C) Fatty acid metabolism, and (D) MYC target pathway scores are plotted by Repair groups and Tumor adjacent normal. (E–G) Gene expression for hepatoblast marker *AFP*, hepatocyte markers *ALB*, and liver metabolomic marker *CYP450* are shown by Repair group. For pairwise comparisons between groups, ns indicates not significant, ** indicates p<0.01 and **** indicates p<0.0001 for all pairwise t-test. included for each measure.

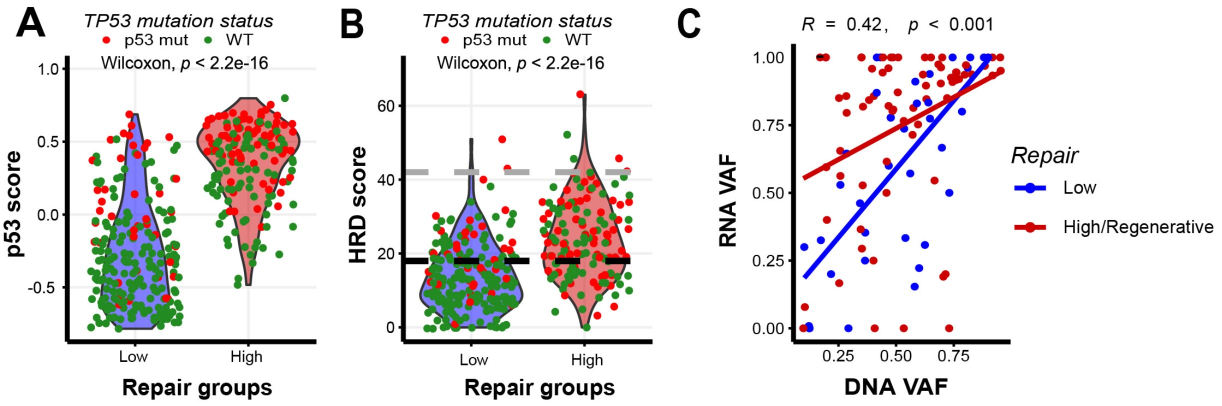

Supplemental Figure S2. ***TP53*** **dysfunction and high HRD are defining features of High repair tumors.** (A) Violin plot of p53 score by Repair groups. Points are colored by *TP53* mutation status, Low repair (blue) and High repair (red); $p < 2.2\text{e-}16$ for Wilcoxon test between repair groups. (B) Violin plot of HRD score by Repair groups. Points are colored by *TP53* mutation status, Low (blue) and High (red); p < 2.2e-16 for Wilcoxon test between repair groups. (C) Variant allele frequencies for *TP53* were calculated from the RNA and DNA sequencing reads. Repair groups are indicated by point color (blue = Low and red = High/Regenerative). Regression lines are plotted and colored according to Repair group.
